# Small extracellular vesicles as key players in cancer development caused by human oncogenic viruses

**DOI:** 10.1186/s13027-022-00471-x

**Published:** 2022-11-28

**Authors:** Shahab Mahmoudvand, Somayeh Shokri, Mohsen Nakhaie, Farid Azizi Jalilian, Ameneh Mehri-Ghahfarrokhi, Reza Yarani, Ali Shojaeian

**Affiliations:** 1 Research Center for Molecular Medicine, Hamadan University of Medical Sciences, Hamadan, Iran; 2 Department of Virology, School of Medicine, Hamadan University of Medical Science, Hamadan, Iran; 3 Gastroenterology and Hepatology, Research Center, Institute of Basic and Clinical Physiology Science, Kerman University of Medical Science, Kerman, Iran; 4 Clinical Research Development Unit, Hajar Hospital, Shahrekord University of Medical Sciences, Shahrekord, Iran; 5 Translational Type 1 Diabetes Research, Department of Clinical Research, Steno Diabetes Center Copenhagen, Gentofte, Denmark; 6 Interventional Regenerative Medicine and Imaging Laboratory, Department of Radiology, Stanford University School of Medicine, Palo Alto, CA USA

**Keywords:** Exosome, Extracellular vesicles, Virus-associated cancer, Human oncogenic viruses

## Abstract

**Background:**

Exosomes are the smallest group of extracellular vesicles in size from 30 to 150 nm, surrounded by a lipid bilayer membrane, and originate from multivesicular bodies secreted by different types of cells, such as virus-infected cells. The critical role of exosomes is information transfer among cells, representing a unique way for intercellular communication via a load of many kinds of molecules, including various signaling proteins and nucleic acids. In this review, we aimed to comprehensively investigate the role of exosomes in promoting human oncogenic viruses-associated cancers.

**Methods:**

Our search was conducted for published researches between 2000 and 2022 by using several international databases includeing Scopus, PubMed, and Web of Science as well as Google scholar. We also reviewed additional evidence from relevant published articles.

**Results:**

It has been shown that exosomes can create the conditions for viral spread in viral infections. Exosome secretion in a human tumor virus can switch on the cell signaling pathways by transferring exosome-encapsulated molecules, including viral oncoproteins, signal transduction molecules, and virus-encoded miRNAs, into various cells.

**Conclusion:**

Given the role of exosomes in viruses-associated cancers, they can also be considered as molecular targets in diagnosis and treatment.

## Introduction

Due to their ability to induce cancers in individuals, infected human oncogenic viruses are responsible for developing several human cancers worldwide [1]. These oncoviruses, as etiological agents for several human malignancies, mainly include Epstein–Barr virus (EBV), hepatitis B virus (HBV), human T-lymphotropic virus 1 (HTLV-1), human papillomaviruses (HPVs), hepatitis C virus (HCV), Kaposi sarcoma-associated herpesvirus (KSHV; also known as human herpesvirus 8 (HHV-8)) and Merkel cell polyomavirus (MCPyV) [2]. It is assumed that in about 15% of infectious cases with oncogenic viruses, various cancers can develop due to the improper functioning of the host immune system in eradicating the virus [3]. Following disruption of normal mechanisms for regulating cell growth and death after oncoviral infection, the cells proliferate uncontrollably, leading to cancer [4]. Most carcinogenic viruses cause chronic infections that last for years without overt symptoms and adapt to the host cell by altering cellular processes and immune system escape mechanisms [5]. Immune system suppression and incomplete immune supervision can compel unlimited viral replication and increase the expression of viral proteins that disorganize the regulation of cell proliferation and induce tumorigenesis. Indeed, the expression of death receptors can be caused by the function of viral oncoproteins and activate extrinsic and intrinsic apoptotic pathways [4]. Recently, it has been shown that the transfer of various bioactive molecules via exosomes derived from oncogenic virus-associated tumors plays a vital role in the improper function of the immune system and remodeling of the cellular microenvironment [6]. Exosomes are extracellular vesicles that were first identified by Harding et al. in 1983 and confirmed by Johnstone et al. in 1987 [7, 8]. This smallest group of extracellular vesicles (30 and 150 nm in diameter) is surrounded by a lipid bilayer membrane and originates from multivesicular bodies secreted by different types of cells, such as virus-infected cells. Depending on the cellular source, state, and environmental conditions, exosomes are highly heterogeneous and dynamic in content, size, and membrane composition [9]. The critical role of exosomes is material trafficking and information transfer among cells, representing a unique way for intercellular communication via load types of molecules, including signaling proteins and nucleic acids [6]. For instance, some oncogenes or proteins are involved in activating tumor-related signaling pathways produced by oncoviruses carried by exosomes [10]. In fact, today, exosomes are considered as a mode of intercellular communication in addition to previously known methods, including direct cell–cell contact or direct transfer of secreted molecules [11, 12].

## The role of exosomes in human oncogenic viruses-associated cancers

**Fig. 1:**
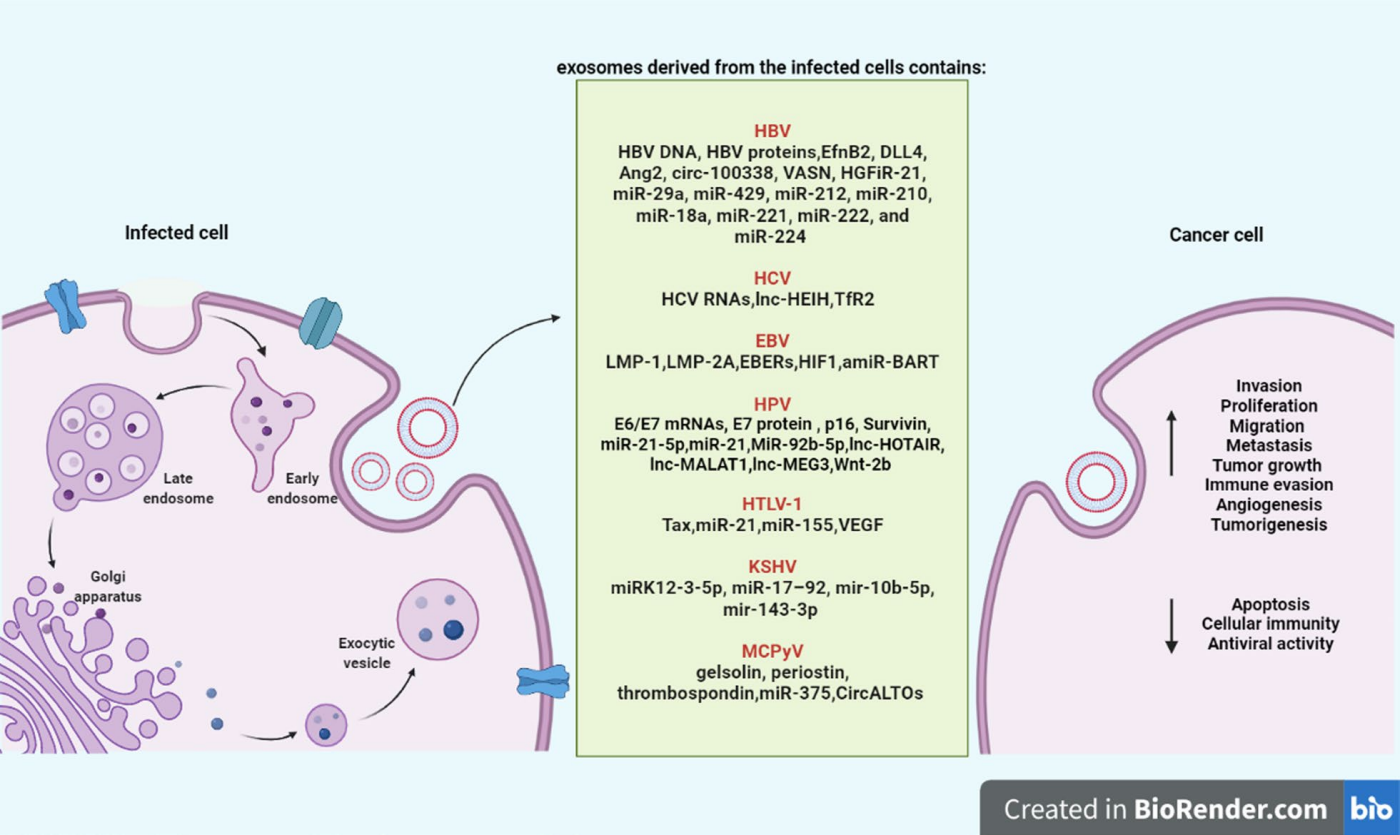
Schematic representation of the mechanism of exosomes during the formation of cancer by viruses. For more details refer to the text

It is now clear that exosomes mediate cell-to-cell communication and biological information exchange via transmitting several molecules such as proteins and various types of nucleic acids (DNA, mRNA, microRNA (miRNA), long non-coding RNA (lncRNA)) between cancer cells and naive cells. In this regard, activation of tumor-related signaling pathways has been shown by some exosome material transported, including oncogenes or related proteins [10]. It has been shown that secretion of exosomes in a human tumor virus can switch on the cell signaling pathways via the transfer of exosome-encapsulated molecules, including viral oncoproteins, signal transduction molecules, and virus-encoded miRNAs into various cells [13]. Although the association between the exosome and the virus is not well established, facilitation and inhibition can be considered two important functions of exosomes in viral infection, leading to the spread of viral and cellular components and inhibiting the immune response. Indeed, exosomes can first create the conditions for spreading and infection [14] (Fig. 1). Then, viruses pursue different mechanisms to pass immune system recognition and survive, and exosomes play an important role in this process. In other words, exosomes derived from oncogenic viruses can interfere with the patient’s immune responses via various functions, leading to remodeling of the cellular microenvironment and creating a favorable microenvironment for accelerating cancer progression and tumorigenesis [15]. Some of this function include facilitate the growth, migration, invasion of malignant cells [2, 13, 16, 17], induce cell apoptosis [18], immune-regulatory [19], decrease anti-viral immune reaction [18], mediate the viral latency [13], promote cellular transformation and cancerization [13, 20], enhance virus-associated cellular transformation [21, 22], control self-replication [23], and alter host cell energy metabolism [20].

### Hepatitis B virus (HBV)

Hepatitis B virus (HBV) is a partially double-stranded circular DNA virus that belongs to the *Hepadnaviridae* family and the *Orthohepadnavirus* genus and is a severe public health problem worldwide [24, 25]. In 2019, the World Health Organization (WHO) estimated that about two billion of the world’s population were living with HBV, with approximately 820,000 million deaths annually [26]. HBV infection can cause both acute and chronic hepatitis B (CHB) and 296 million people are living with chronic infections. CHB can lead to the development of liver cirrhosis and hepatocellular carcinomas (HCCs) [24, 27, 28]. In 2020, HCC is the sixth most prevalent cancer and the third most common cause of cancer deaths worldwide, with about 906,000 new cases and 830,000 deaths annually [29].

Exosomes released by hepatocytes contribute to maintaining proper liver function and are mediators of intracellular communication in the liver cells [30, 31]. In recent years, it has been revealed that exosomes and their contents participate in regulating innate immune responses during HBV infection [32]. Mature HBV virions may bud into multivesicular bodies (MVBs) via an exosome-dependent pathway and significantly improve their transmission through cell-to-cell communication [33]. Increased secretion of extracellular vesicles (EV) is commonly observed in the serum of patients affected by early-stage fibrosis associated with CHB infection [34]. Several studies have demonstrated that exosomes released from the serum of CHB patients carry HBV nucleic acids and HBV proteins [33, 35]. It has been demonstrated that several viral proteins, including HBx, large S, core, and P proteins in exosomes secreted from HBV infected cells, including HepAD38 and Huh-7 cells, facilitate HBV spread [21, 30]. Exosomes containing viral genes derived from HBV-infected hepatocytes led to increased expression of TIR domain-containing adaptor molecule-1 (TICAM-1), natural killer group 2 member D (NKG2D) ligand in macrophages through myeloid differentiation factor 88 (MyD88), and mitochondrial antiviral signaling (MAVS)-dependent pathways, suggesting the importance of exosomes for natural killer (NK) cell activation to produce IFN-γ in the early stage of HBV infection and contribute to HBV clearance during acute HBV infection [33, 36, 37]. It is noteworthy that NK cells play a critical role in controlling HCC [37]. In CHB patients, exosomes can mediate HBV transmission into NK cells by Transforming growth factor-β (TGF-*β*) [35]. Researchers demonstrated that HBV-positive exosomes impair NK-cell functions, including IFN-γ production and the cytolytic activity, resulting in down-regulation of RIG-I in NK cells as well as decreased levels of Nuclear factor kappa B (NF-κB) and p38 mitogen-activated protein kinase (MAPK) [38]. Programmed cell death ligand 1 (PD-L1) is a negative immunomodulatory molecule that inhibits T cell exhaustion, T-cell activation, impairing cytokine production, and inducing the apoptosis of effector T cells [39]. Qun Liu explained the correlation between PD-L1-expression in patients with HCC [40]. Exosomes released by HBV-infected cells are endocytosed by monocytes, causing the upregulation of PD-L1 [33]. CD81, a promoter of tumor growth and metastasis, serves as a coreceptor for viral entry and is reported in exosomes [41]. The antiviral effects of HBV-neutralizing antibodies were diminished by CD81^+^ exosomes, resulting in HBV immune evasion [33]. Hepatocyte growth factor (HGF) is a multifunctional cytokine that activates several downstream signaling pathways to regulate cell proliferation, survival, motility, morphogenesis, differentiation, invasion, angiogenesis, and apoptosis. The HGF plays a role in various biological processes, including liver regeneration and carcinogenesis [42]. Its levels are elevated during active HBV infection and have improved hepatocyte survival [43]. According to Jia et al. study, HGF/MET is secreted from exosomes of HepAD38 cells [30] (Table 1).

**Table 1:** Viral exosome in virus-associated cancer

Virus	Exosome diameter (nm)	Exosome viral	Exosome marker	Targets	Functions	References
HBV	50–100	HBx, large S, core, and P proteins	CD9/CD63	HepAD38, Huh-7	Enhance virus-associated cellular-transformation	[21, 30]
HCV	30–150	NS3/4 protease, E2, Core, genomic RNA	CD9/CD63/CD81, Hsp70/CD63, CD81/AchE, CD63	Treg cells	Transmit HCV and enhance persistent infection, Promote HCV productive infection	[44]
EBV	40–100	miR-BHRF1-3, miR-BART15-3p, miR-BART13-3p, LMP-1, LMP-2A, EBERs	HSC70, flotillin-2, CD9, CD81, CD63	CXCL11, BRUCE, NLRP3, LMP1, ABI2	Immune-regulatory, Induce cell apoptosis, Decrease anti-viral immune reaction, Mediate the viral latency, Facilitate the growth, migration, and invasion of malignant cells	[2, 45, 46]
HPV	50–200	E6/E7, p16, Survivin	Syntenin	p53, pRb	Promote cellular transformation and cancerization	[47, 48]
HTLV-1	70–100	Tax, HBZ, gp61, Env	CD45, CD43	CTLL-2, PBMCs	Cell cycle progression, cell survival, transcription and cell proliferation signaling pathways regulation	[49, 50]
KSHV	50–150	miRK12-3-5p, miR-K12-2-5p, mir-10b-5p, mir-143-3p	CD63	EGLN1,EGLN2,HSPA9	Alter host cell energy metabolism	[51]
MCPyV	30–150	CircALTOs, gelsolin, periostin and thrombospondin, LT-ag	CD63,CD81	MCC, RBPJ, p53	Transcriptional enhancer, increase proliferation and pathogenicity	[52]

Five substances were up-regulated in HCC cell-derived exosomes, including ephrin-B2 (EfnB2), delta-like 4 ligand (DLL4), angiopoietin-2 (Ang2), circ-100338, and Vasorin (VASN), which have been verified to have links with active endothelium-dependent angiogenesis. The notch signaling pathway is crucial for cell proliferation, differentiation, and survival [53]. Two of the Notch signaling pathway components DLL4, a transmembrane ligand for the Notch, and ephrin-B2, a downstream of DLL4/Notch, have recently appeared essential in arterial differentiation of endothelial cells (ECs). Ephrin-B2 and DLL4 could be delivered into endothelial progenitor cells (EPCs) through exosomes to promote tumor angiogenesis [54, 55]. Kongkavitoon et al. showed a link between HBx and DLL4-Notch1 in regulating cell survival in HCC. MEK1/2, phosphatidylinositol 3-kinase (PI3K/AKT), and nuclear factor kappa B (NF-κB) pathways are required for HBx-mediated DLL4 up-regulation [56]. Ang2 is an angiogenic factor that, through its cooperation with the vascular endothelial growth factor (VEGF) pathway, plays a crucial role in angiogenic remodeling and is closely related to the development and prognosis of HCC [57, 58] via the up-regulation of protein kinase B (AKT) endothelial nitric oxide synthase (eNOs) as well as AKT/β-catenin signaling pathways [59]. Sanz-Cameno et al. showed that Ang2 expression was up-regulated at both mRNA and protein levels in the liver of CHB patients [60]. A high exosomal circ-100338 level was detected in high-metastatic HCC cells compared with low metastatic HCC cells. Unlike the abovementioned substances, circ-100338 not only promoted ECs proliferation but increased angiogenesis and cell invasion ability [61]. Several studies observed that circRNA-100338 is a biomarker for prognosis in HBV-related HCC [62–64]. Several studies suggested that serum exosomes and their microRNAs serve as biomarkers for HCC screening and may be related to diseases caused by HBV [65]. In HBV-associated HCC, have shown that increased expression levels of miRNAs in exosomes such as miR-21, miR-29a, miR-429, miR-212, miR-210, miR-18a, miR-221, miR-222, and miR-224. These miRNAs act by dysregulating DNA damage, promoting tumor angiogenesis, and suppressing antiviral activity [66–71]. The endosomal sorting complex required for transport (ESCRT) machinery is involved in several cellular activities, including MVB biogenesis, cellular abscission, and viral budding [72]. Vacuolar protein sorting A (Vps4A), the terminal ATPase of the ESCRT machinery, acts as a tumor suppressor by influencing the microRNA profiles of exosomes and is down-regulated in HCC tissues [73, 74]. In this regard, several studies have shown that the Vps4A is recruited explicitly by the HBV-specific endoplasmic reticulum (ER) [75] and can inhibit HBV replication [76, 77]. A depth understanding of the molecular mechanisms between HBV and HCC may make it possible to predict their outcomes more accurately and give new insights into therapeutic strategies. The role of exosomes suggests that we can modify exosomes for use in HBV treatment.

### Hepatitis C virus (HCV)

Hepatitis C virus (HCV) is an enveloped single-stranded RNA belonging to the *Flaviviridae* family and the *Hepacivirus* genus [78]. HCV infection can be an acute or chronic infection that affects more than 71 million individuals globally [79]. In 2019, the WHO estimated that 58 million people have chronic HCV infection, with approximately 290 000 deaths [80]. Chronic HCV infection causes additional liver damage, such as hepatitis, cirrhosis, and 31% of all HCCs [28]. Exosomes have recently been a viable option for HCV transmission between liver cells [81, 82]. To establish viral infection, exosomes can also successfully transfer HCV RNA to dendritic cells (DCs) [81]. In several studies, exosomes obtained from the plasma of HCV-infected individuals and hepatocyte-derived exosomes contained HCV RNAs [83–85]. Maliha Ashraf Malik et al. confirmed that HCV hijacks the exosomal pathway for the secretion of viral particles [41]. An exciting study has shown that regulatory T (Treg) cells play an essential role in mediating the motility and progression of HCC via inducing the TGF-β1 signaling pathway and epithelial-mesenchymal transition [86]. Exosomes released by hepatocytes after HCV infection can increase the number of Treg cells, thereby reducing the viral immune responses of the body and preventing HCV clearance [82]. Long non-coding RNAs (lncRNAs) are strongly linked to the progression of HCV-related HCC [87]. High expression in hepatocellular carcinoma (HEIH) is a lncRNA that acts as a tumor promoter by accelerating HCC progression [88]. Zhang et al. found that expression of HEIH-lncRNA increased in both serum and exosomes in patients with HCV-related HCC [89]. Inflammation and immunosuppression are among the causes of HCC [90]. Myeloid-derived suppressor cells (MDSCs) play a crucial role in immune response regulation. LncRNA-RUNX1 overlapping RNA (RUNXOR) is associated with the immunosuppression mediated by MDSCs, and RUNXOR expression was negatively correlated with the percentage of T helper type 1 (Th1) and cytotoxic T cells (CTLs) cells in the peripheral blood [91]. HCV-related exosomes induce expressions of RUNXOR and RUNX1 [92].

After HCV infection, transferrin receptor 2 (TfR2) overexpression increases in hepatocytes. Exosomal TfR2 can cause liver iron overload by activating the p38MAPK and extracellular regulated kinase 1/2 (ERK1/2) signaling pathways. In HCV-infected hepatocytes, TfR2 eventually leads to a greater range of cirrhosis and promotes HCC progression [82]. Like HBV, HCV is associated with CD81^+^ in exosomes [41] (Table 1). CD81, a promoter of tumor growth and metastasis, serves as a co-receptor for viral entry. This content suggests that exosomes contribute to the progression of HCC and can also be helpful in the development of novel therapeutic strategies in the future.

### Epstein–Barr virus (EBV)

Epstein–Barr virus (EBV) is a member of the herpes virus family known as human herpesvirus 4 (HHV-4). It is one of the most widespread human viruses, with cases reported worldwide. Many people are infected with EBV while they are young. The most typical way for EBV to spread is through body fluids, particularly saliva. However, it may be transmitted by blood and semen during sexual intercourse, blood transfusions, and organ transplantations [93]. The virus is thought to be present in more than 90% of the world's population, based on epidemiological research [94]. EBV enters a latent phase in B lymphocytes following primary infection after initial infection. The primary infection occurs during asymptomatic childhood, but it could lead to infectious mononucleosis (IM) [95]. Furthermore, other diseases related to this virus include Hodgkin’s lymphoma, Burkitt lymphoma (BL), post-transplant lymphoproliferative disorders (PTLD), nasopharyngeal carcinoma (NPC), and gastric cancer [96]. EBV has a double-stranded DNA genome around 172 kb long and encodes more than 85 genes [97].

In recent years, several works have been done on the role of exosomes in cancer development and dissemination due to their capacity to communicate across cells [2, 98]. It is fascinating that the role of exosomes in EBV infection and persistence has been less well-understood until now. EBV-associated exosomes may be loaded with numerous EBV-related proteins and genes that may contribute to cancer development. According to the available evidence, EBV-associated exosomes carry a range of viral components, including latent membrane protein 1(LMP-1), latent membrane protein 2A (LMP-2A), Epstein–Barr virus-encoded small RNAs (EBERs), viral RNAs, miRNAs that may enhance EBV infection [2, 13, 16, 98–100]. Therefore, it is crucial to understand the biology of exosomes in the pathogenesis of EBV infection. It was found that exosomes have been shown to play a key role in EBV infection and transmission [101, 102]. However, there is little data regarding the roles of EBV product-containing exosomes in cancer development, which have been partly described in several studies [16, 98, 103, 104]. For example, it has been shown that exosomes extracted from EBV-infected NPC cells contain LMP-1 and hypoxia-inducible factor-1a (HIF1a), which play a role in NPC tumor development and progression [2, 99]. Because most EBV-associated exosomes are found in virally infected cells, they might help EBV manipulate the surrounding tumor microenvironment to promote tumor growth and survival. The findings of Ahmed et al., who found that EBV might hijack the exosomal pathway for viral egress and immune evasion, back up this theory [105]. LMP-1 and LMP-2 appear to have an impact on both viral and cancer pathogenesis, according to evidence. The LMP-1 is the most common viral oncogene in EBV-related malignancies and is a potent cancer driver. Exosomes from EBV-infected cancer cells may include this oncoprotein, which means it can be transported from producer to recipient cells through exosomes [2, 13, 106–108]. The exact process by which LMP1 is loaded into exosomes is unknown. In a study conducted by Kobayashi et al., it was found that LMP-1 is linked to ubiquitin C-terminal hydrolase-L1 (UCH-L1) and is directed to the exosomes via C-terminal farnesylation of UCH-L1 [106]. This finding suggestes that inhibiting farnesylation with particular inhibitors limits cancer invasion by blocking exosome-mediated LMP1 transfer during cancer cell-to-cell interactions. Aga et al. illustrated a significant connection between LMP-1 protein levels and the exosomal marker CD63 in NPC tumor tissues, indicating that LMP-1 up-regulates the exosomal release during NPC progression [2]. Because of LMP-1 capacity to activate several cell signaling pathways that promote cell proliferation, angiogenesis, and survival, it is thought to play a key role in NPC pathogenesis. It also impacts antigen presentation, cell–cell interactions, and the production of cytokines and chemokines [109]. According to the findings, exosomes containing LMP-1 may increase EBV pathogenesis via downregulating antiviral immune responses when EBV infection is established in the target cells. It exerts potential immunosuppressive effects to ensure tumor cells proliferate. LMPs play a vital role in suppressing T cell immunological activities by up-regulation of PD-L1 through signal transducer and activator of transcription 3 (STAT3), activator protein 1(AP-1), and NF-κB pathways [110]. In this regard, several studies have shown that exosomes containing LMP-1 derived from EBV-infected lymphoblastoid and NPC cells suppress T lymphocyte activation and inhibit T cell proliferation, which occurs via the inhibitory motif of LMP-1 transmembrane domain [99, 111]. In agreement with this study, Flanagan et al. illustrated that exosomes containing LMP-1 could inhibit peripheral blood mononuclear cells proliferation, suggesting that LMP-1 is involved in immunological regulation. They also showed that in EBV-associated tumors, exosomes containing LMP-1 might be taken up by infiltrating T-lymphocytes and give the anti-proliferative effect of LMP-1. As a result, the tumor cell escapes from the immune system [112]. Another study performed by Mrizak et al. revealed that EBV + exosomes could exert immunosuppressive effects via regulatory T cells (Treg) recruitment into the NPC tumor. They showed that C–C Motif Chemokine Ligand 20 (CCL20) plays an important role in Treg recruitment into NPC tumors [100]. Another work showed that LMP-1 promotes the expression of fibroblast growth factor 2 (FGF-2), as a potent angiogenic factor involved in tumor invasion, in exosomes derived from cells co-transfected with FGF-2 and LMP-1 [113]. In another study, it was observed that LMP-1-containing exosomes from NPC cells could induce active ERK and AKT signaling pathways in the recipient cells [13]. According to studies, ERK and AKT improve cell proliferation or oncogenic transformation [114, 115]. Furthermore, Nanbo et al. showed that exosomes containing LMP-1 derived from type III latency cells upregulate proliferation and intercellular adhesion molecule 1 (ICAM-1) in recipient cells compared to exosomes derived from EBV-negative and type I latency cells [17]. Similarly, the findings show that LMP-1 enhances the release of epidermal growth factor receptor (EGFR) into exosomes, and as a result, these exosomes are taken up by endothelial and epithelial cells, resulting in the activation of ERK and PI3K/Akt pathways [13]. The EGFR is a crucial factor in epithelial cancers, and its activation promotes tumor development, invasion, and metastasis [116]. According to these findings, the effect of LMP-1 on EGFR is a crucial element in EBV-mediated carcinogenesis. Gutzeit and co-workers showed that exosomes derived from the DG75 Burkitt’s lymphoma cell transfected with LMP-1 could be bound to human B cells within peripheral blood mononuclear cells (PBMCs), increasing proliferation and inducing B cells' differentiation into a plasmablast like phenotype [117]. This study illustrated that exosomes containing LMP-1 might influence B cell development. These findings may shed insight on the key role of exosomal LMP-1 in the growth and progression of a variety of EBV-related malignancies. LMP2A was also found to be secreted from cells in exosomes. In EBV-infected cells, LMP2A has been linked to viral latency and pathogenesis [118]. Studies have shown that many oncogenic signaling pathways, including PI3K/Akt, MAPK/ERK, and NF-kB, are activated by LMP2A [119]. Lupus antigen (La) is a widely distributed cellular protein that binds to RNA and is mostly found in the nucleus. Exosomes have also been shown to release this protein outside of the cell. In EBV-infected B cell, EBER binds to La protein in EBV-infected B cells, and since La protein is exported outside the cell via exosomes, EBER has been found in exosomes as well [120]. According to the findings of this research, Baglio et al. reported that cell-to-cell transmission of EBER occurs via exosomes using interacting with La protein and, as a result the virus escapes cytosolic detection in cells [121]. Another study illustrated that exosomes carrying EBER could increase the expression of indoleamine 2,3‑dioxygenase (IDO) in human monocyte‑derived macrophages (MDMs), which in turn might lead to suppression of the proliferation of T lymphocytes and diminish the cytolytic activity of CD8 + T cells [122]. Thus, exosomes containing EBER derived from EBV-infected cells may promote carcinogenesis by suppressing cellular immunity. Exosomes released by infected cells can transport EBV miRNAs. MiR-BamHI A rightward transcript (miR-BART) is one of the most well-known among them. The miR-BART is highly expressed in EBV-related cancers, indicating that it plays a role in carcinogenesis. This miRNA has mostly been studied in NPC, where it is thought to have a role in virus latency, cell proliferation, metastasis, and apoptosis, as well as the control of tumor cell metabolism and immune evasion [123]. The most common miR-BART in NPC is MiR-BART13-3p, which appears to be the best performing NPC-selective marker in diagnostic serum analysis. NPC C666.1 cells secrete a panel of EBV-BARTs via exosomes which miR-BART13-3p is abundantly present in these exosomes [45]. MiR-BART13-3p has been shown to be substantially expressed in NPC patients, particularly those with severe illnesses. Upregulated expression of miR-BART13-3p allows NPC cells to migrate and invade more effectively, resulting in tumor metastasis. It drives epithelial-mesenchymal transition (EMT) by the activating c-JUN/SLUG signaling pathway and targeting ABI2, a tumor suppressor and cell migration inhibitor [46]. Another EBV miRNA that is loaded into exosomes is miR-BART3. MiR-BART3 was abundantly expressed in NPC cells, and it directly targets 3′-untranslated region of tumor suppressor gene DICE1 (DDX26), which led to down-regulation of DICE1 expression as a result to promote cellular growth and cancer development [124, 125] (Table 1). According to the above mentioned, these findings suggest that exosomes contribute to progression of EBV associat-cancer and can be used as diagnostic markers and may help to devise novel therapeutic in the future.

### Human papilloma virus (HPV)

Human papillomavirus (HPV) is the most common sexually transmitted infection, and is the leading cause of cervical cancer. It is well known that nearly 98% of cervical cancers are directly linked to HPV. Cervical cancer is the fourth most diagnosed malignancy in women and the fourth leading cause of cancer mortality. Globally, an estimated 570,000 cases were diagnosed in 2018, leading to 311,000 deaths [126]. The HPVs genome is circular and double-stranded DNA around 8 kb in size and encodes eight genes, two of which have transformative properties (E6 and E7). The oncoproteins E6 and E7 are required for malignant transformation [37]. The E6 protein is hypothesized to stimulate cell proliferation by causing the tumor suppressor protein 53 (p53) to be degraded. The E7 protein attaches to the retinoblastoma tumor suppressor protein (pRb) with a considerably greater affinity, disrupting the connection between Rb and E2 factor (E2F), resulting in the release of E2F factors in their transcriptionally active forms [127]. Until now, the role of exosomes in HPV infection is not clear. Studies have shown that HPV E6/E7 mRNAs are present in exosomes derived from HPV-infected patients [47]. In another study conducted by Kannan et al., it was shown that HPV-16-E7 protein was present in the circulating exosomes of numerous HPV16-associated oropharyngeal cancer cells. They found that the secreted exosomes from these cells containing HPV16-E7 protein increased invasion and induced EMT of non-tumorigenic mammary epithelial cells [20]. There is mounting evidence that HPV E6/E7 oncoproteins significantly impact the miRNA expression of cancer-related cellular miRNAs. These oncoproteins also influence the expression of secretory miRNAs in HPV-infected cells' exosomes [47, 128]. HPV can modify the composition of exosomes, which might have significant ramifications for the intercellular communication of virally transformed cells. In this regard, Honegger et al. showed that HPV E6/E7 oncogene expression silencing significantly impacts the composition and amounts of exosomes released by HPV-positive cancer cells. They investigated the impact of HPV E6/E7 gene silencing on Survivin in HeLa-derived exosomes associated with human cancer development [48]. Ludwig et al*.* reported that exosomes containing HPV isolated from supernatants of the head and neck cancer (HNC) cell lines carried E6/E7, p16, and Survivin [129]. The Survivin has a key role in enhancing cellular proliferation, apoptosis inhibition, survival, and tumor cell invasion, and it can release from cancer cells via exosomes [130]. In a study conducted by Honegger et al., E6/E7 silencing effects on miRNA levels derived from exosomes secreted from HPV-positive cancer cells were evaluated. They illustrated that E6/E7 silencing significantly affects some miRNAs in HPV18-positive HeLa cells via dysregulation of them, which controls cell proliferation and apoptosis. In this study, seven-miRNA were investigated and the result showed that miRNAs let-7d-5p, miR-20a-5p, miR-378a-3p, miR-423-3p, miR-7-5p, miR-92a-3p were downregulated, but miRNA miR-21-5p was up-regulated [131]. Consistent with this study, the result of study conducted by Harden et al. imply that HPV E6/E7 oncoproteins expression changes the expression of miRNAs secreted in exosomes derived from human foreskin keratinocytes (HFKs) [132]. Another study reported that in exosomes isolated from HPV-positive cells, E6/E7 oncoprotein expression can induce the deregulation of expression of some miRNAs involved in tumorigenesis and, as a result, in the tumor cell microenvironment, they can enhance the virus's oncogenic activities [47]. Chiantore et al. discovered that some miRNAs implicated in carcinogenesis such as miR-18a, -19a, -34a and -590-5p were deregulated in K16 and K38 cells infected with HPV16 and HPV38 [133]. MiRNA-21 is involved in cervical cancer oncogenesis. Liu et al. showed that the expression of exosomal miRNA-21 is significantly higher in HPV-positive than HPV-negative normal groups. This suggests that HPV infection can up-regulate of miRNA-21 to develop cervical cancer [134]. To get the carcinogenic property, the miRNA profiles of some exosomes are altered. Wu et al. illustrated that in exosomes isolated from the cervical-vaginal fluid infected with HPV16, some exosomal miRNAs related to the development of cervical cancer were deregulated. In this study, 45 miRNAs are highly upregulated and 55 miRNAs are considerably downregulated after infection with HPV16 compared to the uninfected samples [135]. MiR-92b-5p has been discovered to increase cervical cancer cell proliferation and invasion [136]. Tong et al. found that the expression of miRNA-92b-5p was significantly upregulated in exosomes derived from cells lines infected with HPV [137]. Furthermore, Studies reported that proinflammatory cytokine IL-36γ is present in exosome isolated frompolyinosinic:polycytidylic acid (Poly(I:C))-treated keratinocytes. The poly (I:C)-stimulated expression of some pro-inflammatory genes was reduced by HPV. As a result, HPV may suppress IL-36γ expression in exosomes [138]. It has been made clear that IL-36γ has strong antitumor effects [139]. Zhang et al. showed that exosomal lncRNAs HOTAIR, MALAT1, and MEG3 expression were observed in cervicovaginal lavage samples of cervical cancer-derived exosomes in HPV-positive normal and cancer patients [140]. These lncRNAs are associated with cancer progression, proliferation, metastasis, and invasion [141]. In a study performed by Lin et al., the effect of HeLa cell-derived exosomes on endoplasmic reticulum (ER) stress and tight junctions (TJ) proteins zonula occludens-1 (ZO-1) and Claudin-5 (CLDN5) in human umbilical vein endothelial cell (HUVEC) was evaluated. They found that exosomes derived from HeLa cells promoted metastasis via increased expression of genes involved in the ER stress and decreased the expression of proteins associated with tight junctions ZO-1 and CLDN5, resulting in endothelial integrity breakdown [142]. Liang et al. identified the Wnt-2b protein in exosomes derived from SiHa and HeLa cell lines. They found that fibroblasts take up the exosomes containing Wnt-2b protein and this causes the fibroblasts to be activated and converted to cancer-associated fibroblasts through the Wnt/-catenin signaling pathway, which in turn leads to increasing migration and proliferation of these cells. In this study, it was shown that Wnt-2b protein to be found at high levels in serum exosomes from cervical cancer patients compared to healthy controls [143] (Table 1). With taking everything into consideration, the findings suggest that due to the role of exosomes in HPV related- cervical cancer, they can be used for therapeutic and diagnostic purposes.

### Human T-lymphotropic virus type 1 (HTLV-1)

Human T-lymphotropic virus type 1 (HTLV-1), as the causative pathogen of Adult T-cell leukemia/lymphoma (ATLL), is the first discovered blood-borne human oncogenic retrovirus [144]. This virus is also associated with other progressive diseases such as HTLV-1-associated myelopathy/tropical spastic paraparesis (HAM/TSP) and inflammatory diseases such as HTLV-1-associated uveitis [145]. HTLV-1 encodes different genes, including the structural genes (gag, pro, pol, and env) and non-structural genes (Tax) [146]. HTLV-1 Tax acts as a transactivator and multifunctional protein [147]. It can play a role as the most important virus protein in tumorigenesis by affecting cell cycle progression, cell survival, transcription and cell proliferation signaling pathways regulation, and exosomes and their contents as components in cellular communication [148, 149]. Since exosomes are heterogeneous and differ in size and cargo, knowing their size and contents clarifies their role in pathogenesis and cancer development [49]. Proteomic analysis of exosomes derived from HTLV-1-infected cells in plasma determined that the exosomes are in large quantities (100–200 × 109 particles/mL), have a small size (around 70 nm), and have different cargos [49]. The HTLV-1 takes advantage of exosomes derived from the infected cells by targeting T cells in ATLL, as the virus-infected cells have been shown to secret exosomes containing viral Tax protein [50]. Besides Tax protein, these exosomes can carry multiple hosting factors and viral components, including cellular proteins, mRNA, microRNA, proinflammatory mediators, and viral mRNA transcripts such as Tax, HBZ, gp61, and Env [49, 150]. Tax protein-carrying exosomes promote cell–cell communication and enhance viral spread to other parts, thereby promoting the survival of non-infected cells and exosome-recipient cells against Fas-mediated apoptosis by inducing cFLIP and NF-κB activity [148]. In addition, these exosomes play an important role in increasing the survival of CTLL-2 and PBMCs cells under stress conditions by phosphorylating and activating the AKT and Rb, respectively [148, 150]. Together, these processes, in addition to spreading the virus to more normal cells and facilitating the increase in the pathogenesis of the virus, may provide better conditions for the formation and progression of ATLL via activating the mentioned factors such as NF-κB, AKT, Rb and also preventing apoptosis. The behaviors of ATLL exosomes in cargo transmission are similar to the hormone signaling pathways and act as an endocrine, paracrine and autocrine mechanism [151]. For example, similar to the endocrine mechanism, ATL-derived exosomes carrying Tax and other tumorgenesis-associated factors such as miR-21, miR-155, and vascular endothelial growth factor (VEGF) and their delivery to mesenchymal stem cells (MSCs) can cause changes in their morphology [152]. They can also enhance the MSCs proliferation and increase the expression of those genes involved in migration, such as matrix metalloproteinase-9 (MMP-9) and chemokine receptor type 4 (CXCR-4), and factors involved in VEGF [152].

Moreover, these exosomes can activate the NF-κB pathway in MSCs, and by activating it, its target genes, which are involved in cell proliferation, migration and angiogenesis, are also activated [152]. In the paracrine mechanism, the ATLL EVs probably operate within the tumor microenvironment and can exert their effect on neighboring cells [49, 153]. Although few studies have been conducted on the mechanism of autocrine in HTLV-1-infected cells, a 2015 study by Lavorgna et al. [26] found that Tax oncoprotein stimulates an autocrine loop through IL-17RB-NF-κB signaling and is encapsulated to help self-renewal of infected cells which is essential for leukemogenesis of HTLV-1. Although no drug is approved to prevent the biogenesis and activity of exosomes, approaches based on the manufacture and development of these drugs might provide therapeutic benefits to ATLL patients [49] (Table 1).

### Kaposi’s sarcoma-associated herpesvirus (KSHV)

Kaposi’s sarcoma-associated herpesvirus (KSHV) belongs to the subfamily of *Gammaherpesvirinae* and is also known as human herpesvirus 8 (HHV8) [154]. Due to its association with tumors such as Kaposi Sarcoma (KS) and primary effusion lymphoma (PEL), it is known as an oncovirus [155]. This virus usually initiates its pathogenesis and tumorigenesis by altering the metabolic conditions of infected host cells and neighboring non-infected cells [156, 157]. In this regard, exosomes are one of the favorite tools of gamma herpes viruses. Using them can manipulate the tumor microenvironment through three processes: regulation of downstream signaling pathways, lack of recognition by the immune system, and transformation of cells [158]. Some studies have reported that these viruses have significant impacts on the content of exosomes and their cargo and that these alterations have the potential to spread viral infection, metabolism, translation, migration, and chromatin modeling and enhance tumorigenesis [159]. As KSHV-infected cells mainly express viral genes located within the latency-associated region, it has been found that among these viral genes, 12 viral-encoded miRNAs [160]. These miRNAs are detected from patients' serum, plasma and other body fluids and were packaged inside microvesicles and exosomes for stability [51]. Therefore, miRNAs are considered one of the most important cargos carried by exosomes derived from KSHV-infected cells, including KSHV-encoded miRNAs and host miRNAs [51]. Hoshina et.al’s study showed that almost half the miRNAs (48%) inside the exosomes are KSHV-encoded miRNAs [161], which the highest expression is related to miRK12-3-5p and among the human miRNAs, miR-17–92 cluster, mir-10b-5p and mir-143-3p were reported as the most abundant in exosomes [51, 161]. Some investigations using various methods such as detailed bioinformatics analysis of the genome (Gene ontology) have shown that these exosomal miRNA and oncogenic exosomes can mediate virus pathogenicity by affecting different signal pathways, the metabolic activity of the target cells and other factors such as cytokines and complement system, leading to the development of virus-associated tumors [51, 158, 160]. Aerobic glycolysis or reverse Warburg effect and the Warburg effect mechanism are important metabolic activities affected by the KSHV-encoded miRNAs and exosomal miRNA in infected and uninfected cells, respectively [160]. Induction of aerobic glycolysis and the Warburg effect leads to the survival of infected and non-infected cells in conditions of hypoxia and supports their growth by stimulating the secretion of energy-rich molecules such as lactate and pyruvate [160, 162, 163]. Also, these mechanisms cause the acidification of tumor cell condition by increasing H + ions in the tumor microenvironment, which helps to enhance the invasion of tumor cells [158].

Signaling molecules and pathways are another target of exosomal miRNAs which can facilitate the virus tumorigenesis. The signaling molecules and pathways such as the PI3K/Akt, MAPK, Wnt, TGF-β, focal adhesion and adherens junctions, can be affected by exosomal miRNAs [158]. All these synergically increase KSHV-related tumors [158]. Cytokines disruption is a complex aspect of virus-associated tumorigenesis utilized by exosomes, which eventually promotes malignancy conditions. For example, exosomes can target upstream factors involved in regulating interleukins 6, 10, and 1α, resulting in a decrease in their levels and subsequently reducing inflammation and impairment in recognizing the virus by the immune system [158, 164]. On the other hand, exosomes may cause upregulation in cytokine secretion (such as IL-6) and ultimately enhance cell migration in malignancy conditions [51]. The complement system, another part of the immune response, can be affected by exosomes, playing a role in cancer progression. Investigations have demonstrated that exosomes can contribute to inflammation and the progression of KSHV-associated tumors through activating C3 complement proteins and properdin [165]. Finally, using this communication mechanism (exosomes) by the KSHV, transmitting genetic material, and spreading the infection in non-infected cells causes these cells to remain uninfected and unrecognizable by the immune system [160] (Table 1).

### Merkel cell polyomavirus (MCPyV)

Merkel cell polyomavirus (MCPyV) is a small virus belonging to the *Polyomaviridae* family and is considered a human oncogenic virus [166]. This virus was discovered in 2008 and accounted for the causative role of the virus in the progression of Merkel cell carcinoma (MCC) [166]. MCC is a vital skin malignancy that may be fatal if not treated early due to the high ability of invasion and metastasis of tumor cells [167]. There is no definitive treatment for this cancer, and some treatments, such as immunotherapy to arrest cancer from progressing, have failed [168]. Therefore, recognizing the biomarkers involved in this cancer is urgent and they have been suggested to help MCC screening, early diagnosis, prognosis, and predictive value of MCC [168].

Researches recognize exosomes as one of the essential biomarkers involved in this cancer, the contents of which have been studied using high-throughput approaches and other methods. Various studies have shown that MCPyV–positive MCC cell lines’ exosomes are different in content from MCPyV-negative MCC cell lines’ exosomes, which can affect cancer progression [168]. Cargos loaded inside the exosomes play an essential role in the tumorigenic properties of MCPyV and the spread of its carcinogenesis in normal cells and cancer cells. Some Cargos, which have been evaluated in one study by Ida et al., include the proteins gelsolin, periostin and thrombospondin [169]. As secreted matricellular protein, Periostin is up-regulated in some human cancers and contributes to tumorigenesis through metastasis [169, 170]. Thrombospondin is another matricellular protein released from platelets by stimulating thrombin [169]. Despite the fact that studies have suggested an anti-tumor role for thrombospondin, in some conducted works, invasive properties of human cancer cells is attributed to this element and its high expression in exosomes derived from MCC cells has been mentioned [169, 171, 172]. Gelsolin is a protein that plays a crucial role in regulating the actin filament assembly and disassembly. Several studies have reported its role in increasing the tumorigenesis of oral, lung and colorectal cancers. MCPyV large T-antigen (LT-ag) oncoprotein has been shown to increase MCC by inducing the promoter activity of these three genes and consecutively increasing the expression of these oncoproteins [169]. Moreover, these proteins contribute to tumor expansion by enrichment within the MCC-derived exosomes [169].

As a constant cargo in many exosomes, miRNAs are also present in MCC-derived exosomes and contribute to tumorigenesis by influencing other factors [173]. For example, in a study in 2010, it was found that miR-375 enrichment in MCC-derived exosomes can cause downregulation the expression of two key factors of recombination signal binding protein for immunoglobulin kappa J region (RBPJ) and Tumor protein p53 (TP53) in fibroblast polarization. Low RBPJ and TP53 expression and inducing of fibroblast polarization provide a pro-tumorigenic microenvironment [174]. Circular RNAs (circRNAs) are single-stranded RNAs that, in addition to eukaryotes, can be generated by various oncoviruses, such as EBV, HPVs and MCPyV. These RNAs can show different functions by translating to different proteins [175]. CircRNAs, are another component of exosomes derived from virus-positive Merkel cell carcinoma (VP-MCC) generated by the MCPyV and affect the tumorigenic properties of the virus. It has been reported that circRNAs generated by MCPyV in cell lines and patient tumors encode the alternative large T antigen open reading frame (ALTO) protein as part of the early region [175]. CircALTOs are enriched in exosomes derived from VP-MCC and can function as a transcriptional enhancer and increase proliferation and pathogenicity of MCPyV in surrounding cells and affect genes and pathways related to DNA oncovirus [175] (Table 1).

#### Attractive prospects of exosomes for diagnostic and therapeutic of human oncogenic viruses-associated cancers

Since exosomes do not have the complexity of cells or organs and in contrast, these nanostructures have strengths that make them suitable for treating diseases such as cancer; they attract increasing attention. Some of the strengths mentioned in publications include biocompatibility, immunogenicity, stability, pharmacokinetics, biodistribution, and cellular uptake mechanism, which increase therapeutic index and reduce side effects [176]. In recent years, several studies have highlighted that exosomes, in addition to their various roles in the development and progression of the disease, such as viral-associated cancer, can also be considered a determining molecular target in diagnosis and treatment [177]. Some studies have shown that exosomes derived from oncovirus-infected cells contain some proteins and factors that lead to the activation of signaling pathways (e.g. AKT, ERK and NF-κB) related to the progression of the tumorigenesis [16, 178]. Therefore, decreasing these exosomes may be a therapeutic strategy in oncovirus-associated tumors. On the other hand, the blocking of some proteins (e.g. Galectin-9 in EBV) secreted by oncogenic exosomes may restore the function of immune cells to kill the cancer cells [179].

Furthermore, in exosomes secreted by EBV-infected cells which contain LMP-1, EGFR and PI3K, selective blocking or diminishing exosome diffusion can inhibit the activation of AKT, ERK and NF-κB signaling pathways and tumorigenesis, which present a strategy to suppress the formation of EBV-associated tumors [180]. It has been shown that exosomes originate from umbilical mesenchymal stem cells containing some functional miRNAs that can cause to suppression of viral replication following bind and interaction of miRNA with viral RNA [181]. Exosomes with the promising feature, capable of passing across biological barriers such as the blood–brain barrier, can also be used to develop drug delivery systems as drug vehichle [182]. However, development of exosome based drug delivery strategy necessary to choose the appropriate components to achieve the desired function and efficacy. For instance, engineered exosomes contain HPV-E7 proteins-loaded Nefmut lead to a strong antitumor activity following a protective CD8 ^+^ T cell-mediated immune response [183, 184].

Moreover, Kim et al. showed that the engineered exosomes expressing human leukocyte antigen-A2 stimulate antigen-specific CD8 ^+^ T cells. Therefore, engineered exosomes have appeared as a potential option for cancer therapies, including human oncogenic viruses-associated cancers [185]. Another exciting aspect of exosomes in cancer treatment is the development of the vaccine. Indeed, genetic surface modifications can be made in a peptide located in the transmembrane proteins of exosomes to convey or represent specific antigens [186]. It has shown that the efficiency and bioavailability of some anticancer drugs improved after loading into exosomes. Therefore, it may be a promising therapeutic strategy to prevent virus-associated cancer development [187]. To increase targeting specific cells via exosomes, the cell of origin can be manipulated to express specific genes, which results in engineered cell-derived exosomes loaded with interesting heterologous proteins [31]. Several reports have offered the use of exosomes as the biomarkers in various cancers, allowing earlier detection of cancers and increasing prognosis and survival [188].

## Conclusions

Exosomes play a crucial role in material trafficking, information transferring among cells, and intercellular communication via loading molecules, including various signaling proteins and nucleic acids. It is worth noting that human oncogenic viruses can use exosomes to accelerate viral pathogenesis. A depth understanding of the molecular mechanisms between viruses and cancers may make it possible to predict their outcomes more accurately and give new insights into therapeutic strategies. Together, this content suggests that exosomes contribute to the progression of viruses-associated cancers and can also help develop novel therapeutic strategies in the future. Although there are still many challenges to using exosomes in clinical applications that are critical to be resolved, exosome-based therapies are thought to be a promising avenue for cancer therapy.

## Data Availability

Data sharing not applicable to this article as no datasets were generated or analysed during the current study.

## Abbreviations

EBV: Epstein–Barr virus
HTLV-1: Human T-lymphotropic virus 1
HPV: Human papillomavirus
KSHV: Kaposi sarcoma-associated herpesvirus
HHV-8: Human herpesvirus 8
MCPyV: Merkel cell polyomavirus
miRNA: MicroRNA
lncRNA: Long non-coding RNA
HBV: Hepatitis B virus
WHO: World health organization
CHB: Chronic hepatitis B
HCC: Hepatocellular carcinoma
MVBs: Multivesicular bodies
EV: Extracellular vesicle
NKG2D: Natural killer group 2
MyD88: Myeloid differentiation factor 88
TICAM-1: TIR domain-containing adaptor molecule-1
MAVS: Mitochondrial antiviral signaling
NK: Natural killer
TGF-β: Transforming growth factor-β
STAT3: Signal transducer and activator of transcription 3
AP-1: Activator protein 1
NF-κB: Nuclear factor kappa B
MAPK: Mitogen-activated protein kinase
PD-L1: Programmed cell death ligand 1
HGF: Hepatocyte growth factor
EfnB2: Ephrin-B2
DLL4: Delta-like 4 ligand
Ang2: Angiopoietin-2
VASN: Vasorin
ECs: Endothelial cells
EPCs: Endothelial progenitor cells
VEGF: Vascular endothelial growth factor
eNOs: Endothelial nitric oxide synthase
ER: Endoplasmic reticulum
HCV: Hepatitis C virus
DCs: Dendritic cells
Treg: Regulatory T cells
HEIH: High expression in hepatocellular carcinoma
MDSCs: Myeloid-derived suppressor cells
RUNXOR: LncRNA-RUNX1 overlapping RNA
Th1: T helper type 1
CTLs: Cytotoxic T cells
TfR2: Transferrin receptor 2
IM: Infectious mononucleosis
BL: Burkitt lymphoma
PTLD: Post-transplant lymphoproliferative disorders
NPC: Nasopharyngeal carcinoma
LMP-1: Latent membrane protein 1
LMP-2A: Latent membrane protein 2A
EBERs: Epstein–Barr virus-encoded small RNAs
HIF1a: Hypoxia-inducible factor-1a
UCH-L1: Ubiquitin C-terminal hydrolase-L1
FGF-2: Fibroblast growth factor 2
ERK: Extracellular regulated kinase
AKT: Protein kinase B
ICAM-1: Intercellular adhesion molecule 1
EGFR: Epidermal growth factor receptor
IDO: Indoleamine 2,3‑dioxygenase
MDMs: Monocyte‑derived macrophages
miR-BART: MiRBamHI A rightward transcript
EMT: Epithelial-mesenchymal transition
pRb: Retinoblastoma tumor suppressor protein
E2F: E2 factor
HNC: Head and neck cancer
HFKs: Human foreskin keratinocytes
Poly(I:C): Polyinosinic:polycytidylic
TJ: Tight junctions
ZO-1: Zonula occludens-1
CLDN5: Claudin-5
HUVEC: Human umbilical vein endothelial cells
ATLL: Adult T-cell leukemia/lymphoma
HAM/TSP: HTLV-1-associated myelopathy/tropical spastic paraparesis
MSCs: Mesenchymal stromal/stem cells
MMP-9: Matrix metalloproteinase-9
CXCR-4: Chemokine receptor type 4
PEL: Primary effusion lymphoma
PI3K/Akt: Phosphatidylinositol 3-kinase protein kinase B
LT-ag: Large T-antigen
